# Supplementary material for: Impact of KIR-HLA Genotype on Natural-Killer-Cell-Based Immunotherapy for Preventing Hepatocellular Carcinoma after Living-Donor Liver Transplantation
Source: Cancers (Basel). 2024 Jan 26;16(3):533. doi: 10.3390/cancers16030533 (PMC10854659; doi:10.3390/cancers16030533)
Supplement: Supplementary file 1 [file cancers-16-00533-s001.zip › cancers-2826808-supplementary.pdf]

Table S1
Clinicopathological characteristics of patients in this study

Variables	Radiologically within Milan criteria (MC) n= 66		Radiologically beyond MC n= 11
	Pathologically within MC n= 43	Pathologically beyond MC n= 23	
Age	58 (46- 69)	58 (45- 68)	58 (44- 68)
Gender (male/ female)	32/ 11	14/ 9	8/ 3
Primary disease (viral/ autoimmune/ others)	35/ 2/ 6	21/ 1/ 1	7/ 0/ 4
Blood type imcompatibility (yes/ no)	5/ 38	3/ 20	2/ 9
Donor relation (1st degree relatives/ others)	36/ 7	19/ 4	8/ 3
Immunological desensitization (yes/ no)	5/36	4/ 19	9/ 2
MELD score	13 (7- 37)	12 (6- 21)	15 (6- 31)
Treatment period for HCC before transplantation (year)	0.8 (0- 7.5)	1.8 (0- 9.8)	1.1 (0.1-8.0)
Number of LRT for HCC before transplantation	1 (0- 4)	2 (0- 14)	2 (0- 6)
Des-gamma carboxyprothrombin (AU/ml)	32 (10- 26484)	82 (10- 10950)	78 (10- 640)
Alpha-fetoprotein (ng/ml)	6.3 (1.5- 686.7)	33.9 (3.7- 5384)	18.2 (5- 991.2)
Radiological tumor number	1 (0*- 3)	2 (0*-3)	8 (1- 15)
Radiological tumor diameter (cm)	1.5 (0*- 4.5)	2.3 (0*- 4)	2.5 (1- 8)
Pathological assessment			
Poorly differentiated HCC (-/+)	41/ 2	22/ 1	9/ 2
Pathological tumor number	2 (0**- 3)	6 (1- 47)	5 (0**- 15)
Pathological tumor diameter (cm)	1.6 (0**- 4.5)	2.3 (0.9- 6.3)	3.0 (0**- 8.4)
Pathological microvascular invasion (-/ +)	43/ 0	14/ 9	9/ 2
Adjuvant immunotherapy (-/ +)	27/ 17	9/ 14	4/ 7

* Radiologically suspected to be unviable due to prior loco-reginal therapy
** Defined as complete necrosis by pathological assessment

Table S2
Clinicopathological characteristics of patients receiving the immunotherapy with donor liver-derived NK cells

	With immunotherapy n=38	Without immunotherapy n=39	p value
Age	58 (44- 68)	58 (45- 69)	0.650
Gender (male/ female)	25/ 13	30/ 9	0.279
Primary disease (viral/ autoimmune/ others)	32/2/4	31/1/7	0.557
Blood type imcompatibility (yes/ no)	4/ 34	7/ 32	0.586
Donor relation (1st degree relatives/ others)	32/ 6	31/ 8	0.591
Immunological desensitization (yes/ no)	5/ 33	9/ 30	0.256
MELD score	12 (6-29)	13 (6-37)	0.146
Treatment period for HCC before transplantation (year)	1.1 (0- 5.4)	0.8 (0- 9.8)	0.915
Number of LRT for HCC before transplantation	2 (0- 8)	1 (0- 14)	0.366
Des-gamma carboxyprothrombin (AU/ml)	76.5(10-20805)	31 (10-26484)	0.062
Alpha-fetoprotein (ng/ml)	12.7 (3.2-5384)	6.9 (1.5- 4121)	0.860
Radiological tumor number	2 (0*- 15)	1 (0*- 10)	0.001
Radiological tumor diameter (cm)	2.0(0*- 5.0)	1.7 (0*- 8)	0.191
Radiological Milan criteria (MC) (within/ beyond)	31/7	35/ 4	0.306
Pathological assessment			
Poorly differentiated HCC (-/+)	33/ 5	39/ 0	0.007
Pathological tumor number	3 (0**- 15)	2 (0**- 47)	0.014
Pathological tumor diameter (cm)	2.2 (0**- 6.3)	1.8 (0**- 8.4)	0.116
Pathological microvascular invasion (-/ +)	30/ 8	36/ 3	0.118
Pathological MC (within/ beyond)/Radiologically beyond MC	17/ 14 / 7	26/ 9/ 4	0.149

* Radiologically suspected to be unviable due to prior loco-reginal therapy

** Defined as complete necrosis by pathological assessment

Table S3
Clinicopathological characteristics of patients according to recipient-licensed NK cell genotype

	Recipient poorly licensed NK genotype n= 57	Recipient highly licensed NK genotype n= 20	P value
Age	60 (50- 68)	57 (44- 69)	0.14
Gender (male/ female)	43/ 14	12/ 8	0.20
Primary disease (viral/ autoimmune/ others)	45/ 3 / 9	18/ 0 / 2	0.30
Blood type imcompatibility (yes/ no)	47/ 10	19/ 1	0.13
Donor relation (1st degree relatives/ others)	45/ 12	18/ 2	0.25
Immunological desensitization (yes/ no)	11/ 46	3/ 17	0.66
MELD score	13 (6- 37)	13 (7 -31)	0.57
Treatment period for HCC before transplantation (year)	0.82 (0.10- 8.34)	1.37 (0- 9.80)	0.94
Number of LRT for HCC before transplantation	1 (0-9)	1 (0-14)	0.51
Des-gamma carboxyprothrombin (AU/ml)	43 (10-4678)	70 (12-26484)	0.25
Alpha-fetoprotein (ng/ml)	5 (1.5- 991.2)	23.9 (3,1- 5384)	0.09
Radiological tumor number	1 (0*-15)	2 (0*-8)	0.62
Radiological tumor diameter (cm)	2 (0*-5)	1.68 (0*-8)	0.62
Adjuvant immunotherapy (-/ +)	28/ 29	11/ 9	0.65
Pathological assessment			
Poorly differentiated HCC (-/+)	53/ 4	19/ 1	0.75
Pathological tumor number	3 (0**-37)	2 (0**-47)	0.10
Pathological tumor diameter (cm)	2.1 (0**-6.3)	1.55 (0**-8.4)	0.27
Pathological microvascular invasion (-/ +)	50/ 7	16/ 4	0.41

* Radiologically suspected to be unviable due to prior loco-reginal therapy
** Defined as complete necrosis by pathological assessment

Table S4
Tumor characteristics and KIR-HLA combination genotype in patients at risk of HCC recurrence

			Prognosis	HLA-KIR genotype assessment			Preoperative assessment				Pathological assessment			
		Immuno -therapy	Time to recurrence (year)	Recipient licensed NK genotype	Donor licensed NK status	Number of receptor-ligand mismatch	Radiological tumor number	Radiological tumor diameter (cm)	AFP ng/ml	DPC AU/ml	Histological diagnosis	Pathological tumor number	Pathological tumor diameter (cm)	Micro vascular invasion
Pathologically beyond Milan criteia	#1	-	0.7	Highly	Highly	2	1	2.7	917.5	13	Mod	1	3.7	+
	#2	-	2.5	Poorly	Highly	2	0	0	13.4	98	Mod	4	1.8	-
	#3	-	1.2	Highly	Poorly	1	1	1.8	4121	130	Mod	47	1.0	-
	#4	-	NR	Poorly	Poorly	2	1	1.0	5.6	12	Mod	25	0.9	-
	#5	-	NR	Poorly	Poorly	2	3	2.5	6.5	31	Mod	6	2.3	-
	#6	-	NR	Poorly	Poorly	2	2	2.3	33.9	11	Mod	37	3.2	-
	#7	-	NR	Poorly	Poorly	2	1	4.0	43.5	858	Mod	3	2.9	+
	#8	-	NR	Poorly	Poorly	2	3	1.0	44.8	4678	Mod	10	2.1	-
	#9	-	NR	Poorly	Poorly	2	1	2.0	128.7	26	Mod	4	2.0	-
	#10	+	NR	Highly	Highly	1	2	2.9	2578	24	Mod	2	4.5	-
	#11	+	NR	Poorly	Highly	2	1	3.3	4.2	10	Mod	7	3.0	-
	#12	+	NR	Poorly	Highly	2	2	1.0	231.4	110	Mod	7	1.4	-
	#13	+	NR	Highly	Poorly	1	3	2.6	11.6	10950	Por	3	2.5	+
	#14	+	NR	Highly	Poorly	1	3	2.8	32.8	3026	Mod	2	4.0	+
	#15	+	1	Highly	Poorly	1	3	1.7	5384	545	Mod	13	2.2	+
	#16	+	NR	Poorly	Poorly	2	2	3.0	5	54	Mod	9	2.9	-
	#17	+	NR	Poorly	Poorly	3	1	1.0	5	55	Mod	1	1.3	+
	#18	+	1.5	Poorly	Poorly	2	3	0.5	9.9	415	Mod	2	6.3	+
	#19	+	NR	Poorly	Poorly	2	2	3.0	11.2	13	Mod	4	3.3	-
	#20	+	NR	Poorly	Poorly	2	1	1.8	19.8	355	Mod	8	1.6	-
	#21	+	NR	Poorly	Poorly	2	2	2.0	36.5	17	Mod	7	1.9	-
	#22	+	NR	Poorly	Poorly	2	3	2.5	248.9	580	Mod	6	2.4	+
	#23	+	NR	Poorly	Poorly	2	3	2.3	251	82	Mod	5	2.2	+
Radiollogically beyond Milan criteia	#24	-	NR	Poorly	Highly	2	8	2.0	102.4	67	Mod	8	3.0	-
	#25	-	2.7	Highly	Poorly	2	1	8.0	7.5	25	Mod	1	8.4	-
	#26	-	0.9	Poorly	Poorly	2	1	2.5	735	10	Mod	5	2.0	+
	#27	-	NR	Poorly	Poorly	2	10	1.6	5	17	Necrosis	0	0.0	-
	#28	+	2.1	Highly	Highly	1	8	1.5	81.3	78	Well	10	1.5	-
	#29	+	3.1	Poorly	Highly	2	1	2.0	5.5	304	Por	11	2.2	-
	#30	+	NR	Poorly	Highly	2	10	2.2	53.2	928	Mod	15	3.5	-
	#31	+	1.8	Poorly	Poorly	2	3	5.0	5.4	157	Mod	7	4.0	-
	#32	+	0.9	Poorly	Poorly	2	8	3.0	5	640	Mod	5	3.5	-
	#33	+	NR	Poorly	Poorly	2	2	3.8	18.2	150	Mod	2	3.5	-
	#34	+	NR	Poorly	Poorly	2	15	2.6	991.2	75	Por	10	2.9	+

Figure S1

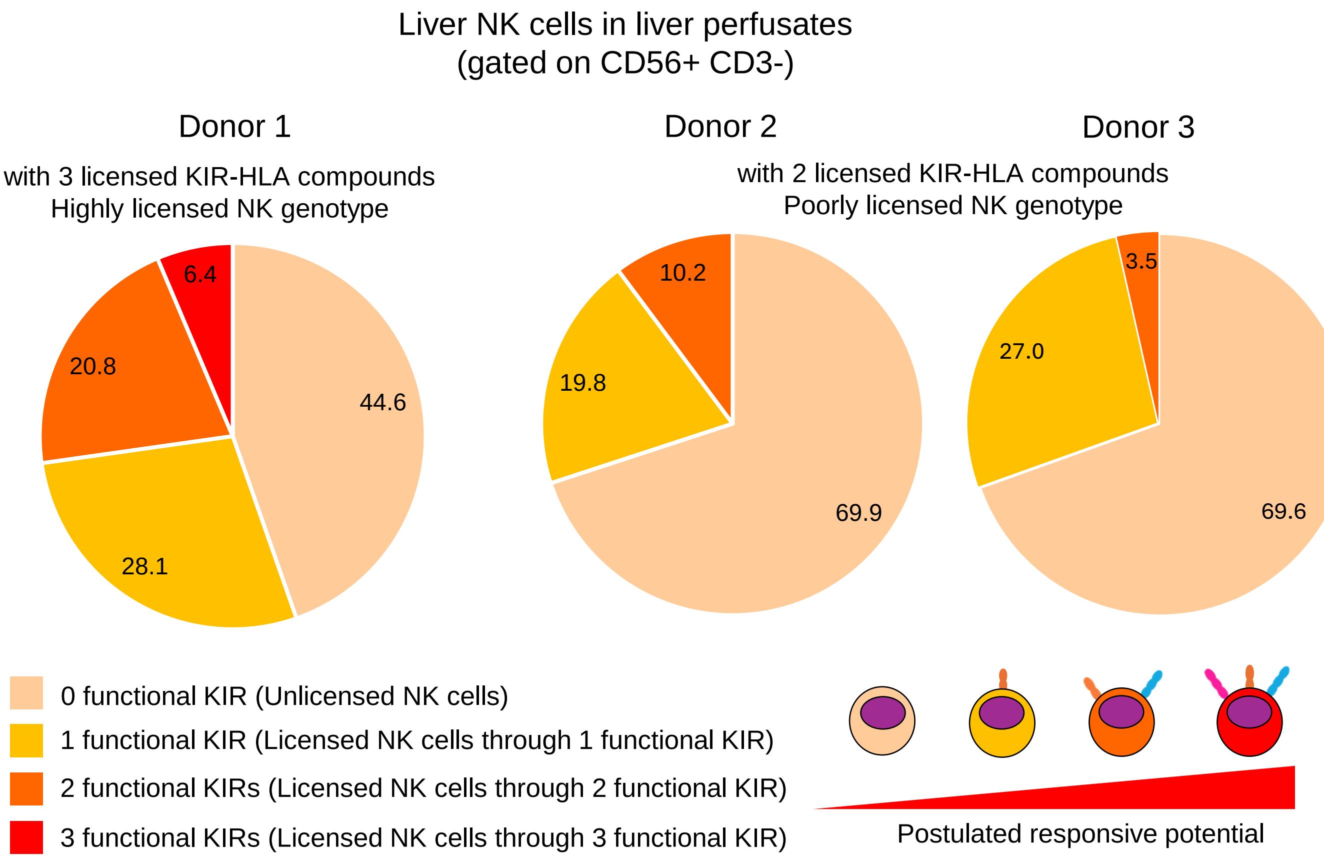

Figure S1; Liver NK cells from the donor with highly licensed NK genotype were enriched with highly licensed NK cells in KIR expression level

Multi-color flow cytometry analysis revealed that liver NK cells from the donor with highly licensed NK genotype (donor 1) was enriched with highly licensed NK cells confirmed by KIR expression level compared with those from poorly licensed NK genotype (donor 2 and 3).

Figure S2

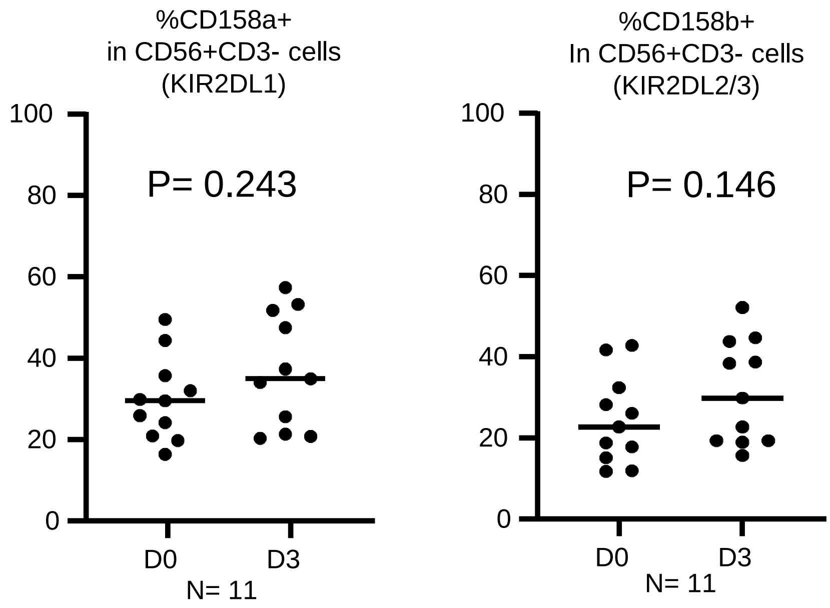

Figure S2; KIR expression in liver NK cells was preserved after 3 days of stimulation with IL-2.
Dot plots showed the % positive of CD158a (left) and CD158b (right) in CD56+ CD3- NK cells before (D0) and after 3 days IL-2 activation (D3) assessed by flow cytometry. Statistical analyses were performed by Wilcoxon tests.
